# The impact of biomembranes and their dynamics on organismic aging: insights from a fungal aging model

**DOI:** 10.3389/fragi.2024.1356697

**Published:** 2024-01-24

**Authors:** Heinz D. Osiewacz

**Affiliations:** Institute for Molecular Biosciences, Faculty of Biosciences, Goethe University, Frankfurt, Germany

**Keywords:** aging, *Podospora anserina*, biomembranes, phospholipids, membrane trafficking, mitochondria, vacuoles, autophagy

## Abstract

Biomembranes fulfill several essential functions. They delimitate cells and control the exchange of compounds between cells and the environment. They generate specialized cellular reaction spaces, house functional units such as the respiratory chain (RC), and are involved in content trafficking. Biomembranes are dynamic and able to adjust their properties to changing conditions and requirements. An example is the inner mitochondrial membrane (IMM), which houses the RC involved in the formation of adenosine triphosphate (ATP) and the superoxide anion as a reactive oxygen species (ROS). The IMM forms a characteristic ultrastructure that can adapt to changing physiological situations. In the fungal aging model *Podospora anserina*, characteristic age-related changes of the mitochondrial ultrastructure occur. More recently, the impact of membranes on aging was extended to membranes involved in autophagy, an important pathway involved in cellular quality control (QC). Moreover, the effect of oleic acid on the lifespan was linked to basic biochemical processes and the function of membranes, providing perspectives for the elucidation of the mechanistic effects of this nutritional component, which positively affects human health and aging.

## 1 Introduction

Aging of biological systems is a complex process leading to functional degeneration and, ultimately, the death of the system. It is under the control of genetic, environmental, and stochastic factors. Over the many years of intensive research using different biological systems ranging from unicellular organisms to the human species, several aging hallmarks such as molecular damage, genetic instabilities, impairments in repair and degradation, telomere shortening, and mitochondrial dysfunction were identified (López-Otín et al., 2013; Kennedy et al., 2014; Szklarczyk et al., 2014).

**FIGURE 1:**
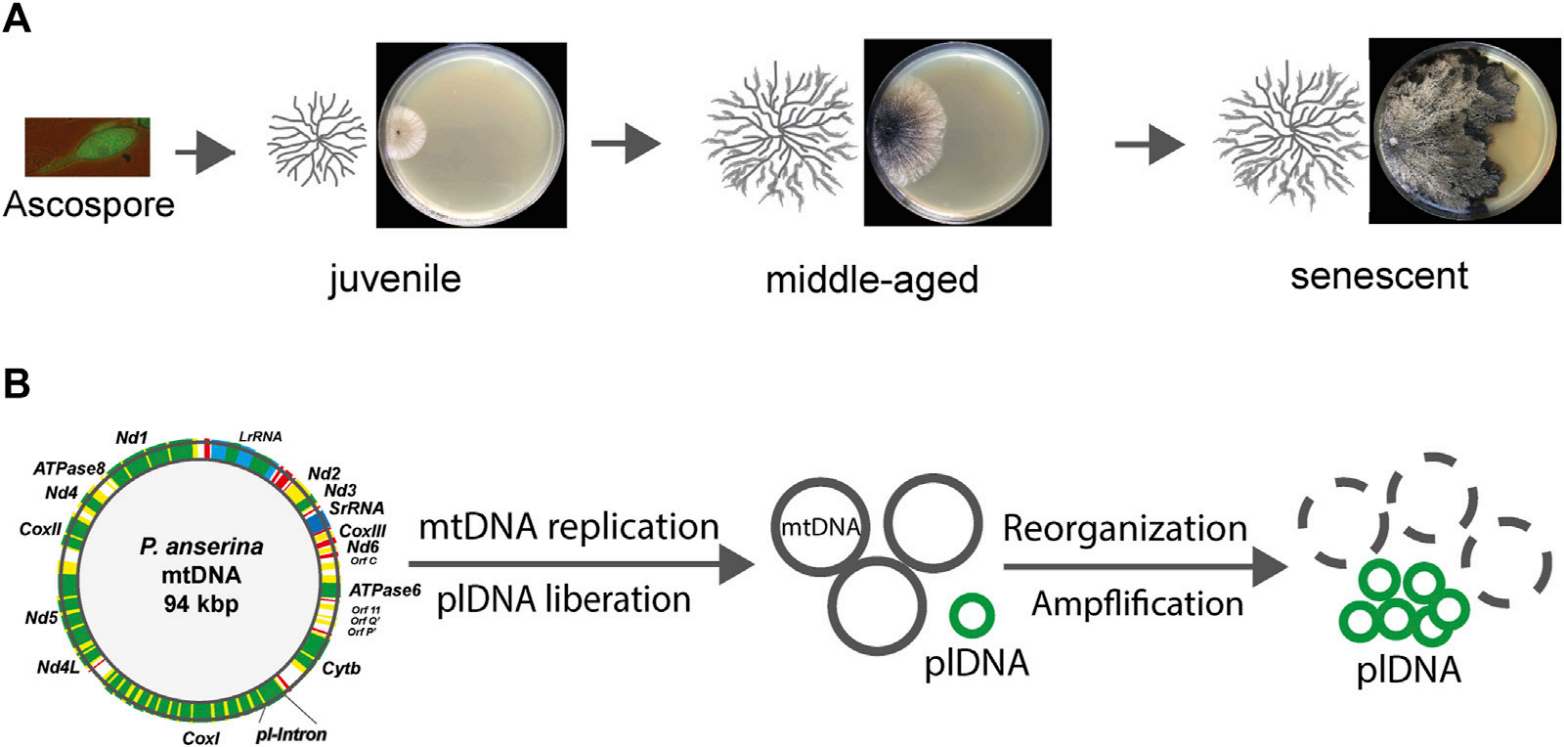
Age-related reorganization of mtDNA during aging of *Podospora anserina*. **(A)** Starting from a unicellular ascospore, the product of sexual reproduction, a juvenile mycelium develops that grows at the periphery. After a strain-specific period of growth, it reaches a senescent stage, at which growth ceases and ultimately stops. Finally, the filamentous cells at the periphery burst. The figure shows a developing mycelium schematically (left) on an agar plate containing a solid growth substrate. **(B)** Schematical alterations of the mtDNA. In juvenile cultures, the mtDNA is a circular molecule of 94 kbp, with 13 genes (green) encoding respiratory chain proteins, clusters coding mitochondrial tRNAs (red), and two rRNA genes (blue). The coding sequences of some genes are interrupted by intron sequences (yellow). During aging, the first intron (pl-intron) of cytochrome c oxidase gene subunit I (*CoxI*) is liberated and amplified (plDNA). This molecule acts a mutator and leads to the deletion of large parts of the mtDNA.

*Podospora anserina* is a multicellular filamentous ascomycete closely related to the unicellular ascomycete *Saccharomyces cerevisiae* (baker’s yeast). The life cycle of *P. anserina* starts with the germination of an ascospore, the product of sexual reproduction, and the formation of a vegetation body (mycelium) that consists of a network of branched filamentous cells called hyphae, which grow on their tips (Figure 1). After a strain-specific growth period, the hyphal tip growth slows down until it comes to a complete stop, and the hyphae burst at their tips (Rizet, 1953). *P. anserina* was intensively used as an experimental model to unravel the molecular basis of organismic aging, and a strong mitochondrial etiology of aging was demonstrated early. Most importantly, the reorganization of the mitochondrial DNA (mtDNA) during the lifespan was found to lead to molecular degeneration, causing the death of the system. The stabilization of mtDNA was found to be a key to extending the lifespan (Osiewacz et al., 1989). Other specific genetic manipulations, including those lowering the cellular load of reactive oxygen species (ROS) or improving molecular pathways involved in mitochondrial quality control, were uncovered as being effective in increasing the lifespan of *P. anserina*. The various experimental studies are discussed in different comprehensive reviews (Esser and Tudzynski, 1980; Osiewacz, 2002; Scheckhuber and Osiewacz, 2008; Hamann and Osiewacz, 2018; Osiewacz and Schürmanns, 2021).

In this review, I will focus on aspects from which an important role of biomembranes and their building blocks in aging emerged. I will first summarize the early work that uncovered the important role of the IMM, which houses the RC as a key protein complex to carry out the bioenergetic function of mitochondria. Next, I will discuss age-related dynamic changes in the mitochondrial architecture and morphology and the role of biomembranes in mitochondrial quality control. Finally, I will deal with recent studies linking cellular membrane fluxes and metabolic responses to alterations in nutrition. These rather new findings provide perspectives for more detailed investigations, including careful translational research to test the situation in diverse organisms, including the complex human species with its many different organs, tissues, and cells.

## 2 Respiration

Soon after the first description of *P. anserina* aging in 1953, genetic investigations revealed an extrachromosomal basis of this process (Rizet, 1953; Marcou, 1961). Subsequently, mitochondria were identified to carry the corresponding genetic traits. Specifically, it was found that the mitochondrial DNA (mtDNA) becomes reorganized during aging (Figure 1). A part of this DNA, which was later demonstrated to exactly correspond to the first intron in the gene coding for subunit I of cytochrome c oxidase (COXI), is liberated from mtDNA and becomes amplified. This DNA is a covalently closed circular DNA resembling the structure of bacterial plasmids and, therefore, was termed plasmid-like DNA (plDNA) or, due to its accumulation in senescent cultures, αsen DNA (Stahl et al., 1978; Cummings et al., 1979; Kück et al., 1981; Osiewacz and Esser, 1984). This genetic element behaves like a mutator and is able to reintegrate into the mtDNA, generating large sequence duplications between which genetic reorganizations can occur (Sellem et al., 1993). As a consequence, large parts of the mtDNA are deleted during aging (Kück et al., 1985) (Figure 1). The mtDNA encodes 13 essential proteins of three RC complexes (I, III, and IV) and the ATP-synthase complex, where most of the cellular energy unit ATP is generated by oxidative phosphorylation (OXPHOS). RC complex II is exclusively encoded by the nuclear DNA. In addition, a set of tRNAs and two rRNAs are encoded by the mtDNA.

In addition to ATP, the RC generates superoxide anions (in short, superoxide), charged ROS that cannot directly cross the phospholipid bilayers of membranes. Superoxide can be converted to hydrogen peroxide, which is uncharged and membrane-permeable (Fridovich, 1999; Sies, 2017; Osiewacz and Schürmanns, 2021). Hydrogen peroxide can be detoxified to water by peroxidases or catalase. Alternatively, in the presence of copper (I) and iron (II), hydrogen peroxide can give rise to the formation of the hydroxyl radical, which is highly reactive and very effective in causing damage to all kinds of molecules in its immediate neighborhood. For this ROS, no enzymatic detoxification system exists. Overall, the activity of the RC is both essential and potentially dangerous.

Respiration in *P. anserina* is very flexible. Impairments of individual components of the RC can be overcome by the induction of genes coding for alternative RC components. Such cellular responses provided very important insights into the role of mitochondria in lifespan control. For example, in mutants such as ex1 and ex2, large parts of the mtDNA, including the pl-intron, are deleted (Kück et al., 1985; Schulte et al., 1988; Schulte et al., 1989). These mutants appear to be immortal since they were cultivated for more than 20 years instead of only a few weeks as the wild type without expressing the senescence syndrome. Due to the deletion of the gene encoding cytochrome c oxidase subunit I (COXI), standard COX-dependent respiration is not possible. Instead, respiration proceeds via an alternative terminal oxidase (AOX), which is integrated into the inner mitochondrial membrane (IMM). The gene encoding this protein is induced in a variety of RC mutants of *P. anserina*. The AOX accepts electrons from the ubiquinol pool in the IMM and transfers them directly to oxygen. Following this route, complex III, which is a major generator of superoxide, and complex IV of the cytochrome c-dependent standard RC are bypassed. As a consequence, less ROS are generated. Since ROS are the main contributors to the aging process, the process of the corresponding mutants is altered. The exact way in which ROS act in the control of aging is still a matter of debate (Harman, 1972; Zintel et al., 2010; Hekimi et al., 2011; Barja, 2019). In *P. anserina*, a strong impact of ROS generation by the RC was repeatedly demonstrated (Borghouts et al., 2002a; Borghouts et al., 2002b; Gredilla et al., 2006; Scheckhuber et al., 2011).

## 3 Mitochondrial ultrastructure

**FIGURE 2:**
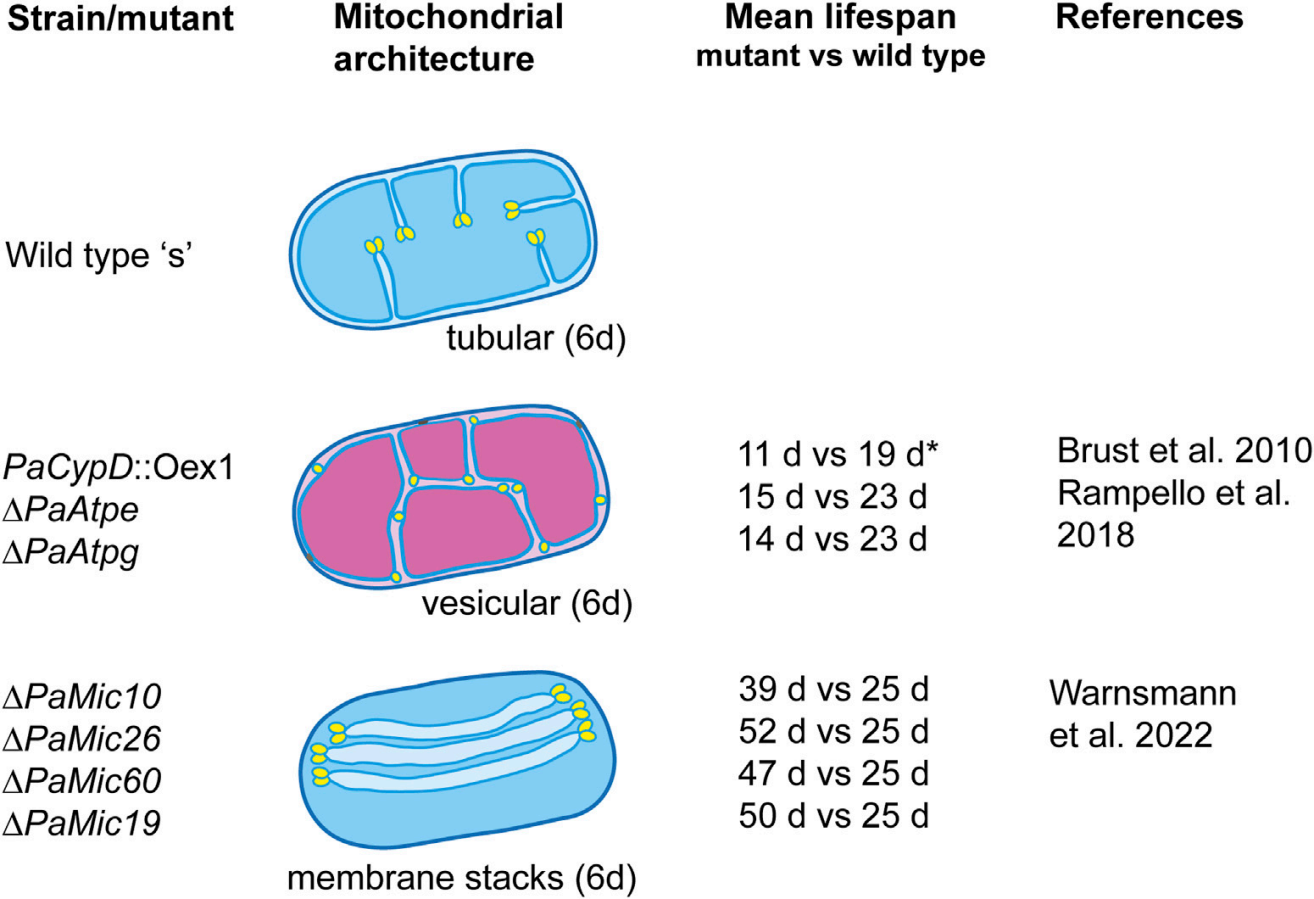
Architecture of mitochondria in 6-day-old (juvenile) and 20-day-old (senescent) mycelia of the wild-type “s” and of selected lifespan mutants of *Podospora anserina.* The altered ultrastructure has a pronounced effect on the mitochondrial function and lifespan. Functional mitochondria are indicated in blue. Functionally impaired mitochondria are indicated in magenta. The mean lifespan of each mutant is compared to the lifespan of the wild type grown under the same growth conditions (i.e., medium and incubation temperature). All strains, except *PaCypD*_Oex1 (grown on the PASM medium), were grown on the standard M2 medium. The medium composition is described in the indicated references.

The aging of *P. anserina* is strongly linked to the activity of the RC, which is localized in the specialized invaginations of the IMM called cristae. The architecture of this part of the IMM, the cristal membrane (CM), is established by protein complexes and phospholipids. In *P. anserina*, pronounced alterations of the ultrastructure occur during wild-type aging. These changes can be induced by specific experimental interventions (Figure 2).

The first evidence of the impact of the mitochondrial ultrastructure on aging was observed in a mutant in which the gene coding for cyclophilin D (PaCYPD), a peptidyl prolyl-cis, trans-isomerase that increases in abundance during aging of the *P. anserina* wild type (Groebe et al., 2007), was overexpressed (Brust et al., 2010). The protein is involved in the opening of the mitochondrial permeability transition pore (mPTP), a large protein complex of unsolved biochemical structure (Halestrap, 2005; Bernardi et al., 2023), which is located in the IMM and is active in the induction of programmed cell death (PCD). The *PaCypD* overexpressor mutant is characterized by a much reduced lifespan. In comparison to the wild type, the mean lifespan of two independent overexpressors was reduced by 61% and 50%, respectively. Most remarkably, in mutant cultures that are 6 days old, a young age in the wild type, the mitochondrial ultrastructure is already like that in senescent mitochondria. Instead of containing tubular cristae, the mitochondrial matrix is filled with vesicles (Brust et al., 2010). A thorough analysis of wild-type mitochondria from cultures of different ages unraveled the dynamic reorganization of the IMM that occurs during aging (Daum et al., 2013). Mitochondria from young cultures contain typical lamellar cristae reaching out into the matrix (Figure 2). At the tips of the cristae, rows of F_1_F_o_-ATP-synthase dimers are located. At the cristae base, the cristae junction (CJ), large protein complexes called the “mitochondrial contact site and cristae organization system” (MICOS), consisting of an MIC10 and MIC60 subcomplex, are involved in negative curvature formation and in establishing contact of the IMM with the outer mitochondrial membrane (OMM) (Marschall et al., 2022; Warnsmann et al., 2022). During wild-type aging, it was shown that the F_1_F_o_-ATP-synthase dimers dissociate, the tubular cristae recede, and finally, the IMM forms a vesicular (reticular) system of membranes filling the mitochondrial matrix space. Moreover, the IMM and OMM were occasionally found to be in close contact and connected by an electron-dense material of unknown composition. At these contact sites, the OMM can rupture, releasing the matrix vesicle into the cytoplasm. This release of mitochondrial material induces PCD, which in *P. anserina* is executed by metacaspases and mitochondrial components such as apoptosis-inducing factor 1 (PaAIP1) (Hamann et al., 2007; Hamann et al., 2008; Strobel and Osiewacz, 2013; Minina et al., 2020).

The critical impact of the F_1_F_o_-ATP-synthase dimers and mitochondrial ultrastructure on aging was further underlined by a study of two mutants in which the two assembly factors PaATPE and PaATPG of the ATP-synthase dimers complex were ablated (Figure 2). Mitochondria from 6-day-old cultures of these mutants are characterized by a vesicular ultrastructure, impaired mitochondrial function, and a mean lifespan that is reduced by 33% and 42%, respectively (Rampello et al., 2018).

The age-related remodeling from lamellar cristae to a reticulate inner membrane system requires changes at the base of the cristae, the cristae junctions, and the MICOS. Two recent studies unraveled this impact with some unexpected and surprising results. The ablation of four of the five identified proteins with homology to yeast MIC components resulted in changes of the mitochondrial architecture, affected mitochondrial functions, and caused a pronounced increase of 56%–108% of the mean wild-type lifespan. The majority of mitochondria from *PaMic10*-, *PaMic19*-, *PaMic60*-, and *PaMic26*-deleted strains contain stacks of floating tubular cristae that are not connected to the inner boundary membrane (IBM), the part of the IMM connected to the CM (Figure 2). The *PaMic12*-deletion strain an exception with wild-type-like cristae, filamentous mitochondria, and a mean lifespan that does not differ from that of the wild type (Warnsmann et al., 2022). Each of the free-floating tubular membranes contains two tips generated by the convex membrane curvature. Although not firmly shown yet, this curvature is likely to be generated by rows of F_1_F_o_-ATP-synthase dimers, as they were demonstrated at the tips of typical wild-type cristae that are connected to the IBM (Davies et al., 2012; Daum et al., 2013). This situation may lead to strong energetic consequences that contribute to the unexpected lifespan extension of the mutants.

The MICOS contains two subcomplexes, MIC10 and MIC60. In *P. anserina*, three genes encoding homologs of PaMIC10 and its putative regulator proteins PaMIC26 and PaMIC27 were identified. A MIC12 homolog of the yeast MIC10 subcomplex seems to be missing in *P. anserina*. Oligomerization of MIC10 is known to lead to the concave curvature of the IMM at the cristae junctions. The MIC60 subcomplex is constituted by two proteins, MIC60 and its regulatory subunit MIC19, which are known to form contact sites between the IMM and the OMM in yeast and mammals. The homologs of *P. anserina* are PaMIC60 and PaMIC19, respectively. Simultaneous deletion of the *PaMic10* and *PaMic60* gene resulted in a synergistical lifespan increase, indicating that the two MIC subcomplexes affect two independent pathways that impact aging. Subsequent analyses revealed that the lifespan-extending effect of PaMIC10 mutants results from a mild induction of oxidative stress leading to mitohormesis, an adaptive response, as it is also known in other organisms such as *Caenorhabditis elegans* (Maglioni et al., 2014; Schiavi et al., 2015), which are beneficial for the organism and may lead to an increased lifespan (Ristow and Zarse, 2010; Yun and Finkel, 2014; Barcena et al., 2018). This conclusion is supported by the addition of the antioxidant ascorbic acid to the growth medium, which reverts longevity of the *PaMic10* and *PaMic26* mutants. Moreover, while 80 μM of the superoxide generator paraquat increased the lifespan of the wild type, it decreased the lifespan of the two *PaMic10* subcomplex-deleted mutants. This suggests that in these mutants, the basic ROS levels were higher than in the wild type, and exogenous paraquat increased the ROS levels beyond beneficial and hormetic levels. In contrast, the effect on the lifespan of the *PaMic60* subcomplex mutants and the wild type by paraquat did not differ, and ascorbic acid added to the growth medium decreased the lifespan of the mutants to the wild-type levels (Warnsmann et al., 2022). This suggests that the lifespan-increasing effect in *PaMic60* subcomplex mutants is not due to a ROS-dependent mitohormetic response.

A lipidomic analysis revealed that the mitochondrial phospholipid profile and the acyl composition of the mitochondrial signature phospholipid cardiolipin (CL) differ from those of wild-type mitochondria. CL is a phospholipid synthesized in the mitochondria by cardiolipin synthase (CRD) as premature CL (pCL) that subsequently is remodeled to monolysocardiolipin (MMCL) by phospholipase and finally by trans-acylase to mature CL (mCL). In *P*. *anserina*, CRD and phospholipid homeostasis are regulated by the IMM-bound protease PaIAP, which adapts membrane plasticity in response to natural fluctuations of environmental conditions (i.e., growth temperature) (Löser et al., 2021). Depending on the number and kind of acyl residues, different forms of CL exist. In the mitochondria of the *PaMic10* and *PaMic26* mutants, compared to the wild type, CL72:8, which contains four linoleic acids, was found to be increased. In mammals, this CL was linked to enhanced mitochondrial function (Stefanyk et al., 2010; Oemer et al., 2020; Oemer et al., 2021). In addition to its impact on the enzymatic functions (e.g., of the RC), the cone-shaped IMM phospholipid CL contributes to membrane curvature formation. Thus, the changed phospholipid composition found in *PaMic60*-mutants is linked to changes in ultrastructure, affects mitochondrial membrane fluidity and enzyme activity, and contributes to the observed increase in lifespan.

## 4 Mitochondrial morphology

Starting from a single ascospore of *P. anserina*, there develops a multicellular vegetation body that requires an increase in the number and distribution of mitochondria. This process proceeds via the incorporation of the membrane material and of proteins into the IMM and OMM of the existing mitochondria. In this way, small ellipsoid mitochondria grow to filamentous units, which subsequently may divide. Depending on the physiological situations, individual mitochondria can also fuse to form larger filaments or branched filamentous networks. Mitochondrial fission and fusion are processes depending on the dynamic reorganization of the two mitochondrial membranes. They are controlled by nuclear encoded proteins (Shaw and Nunnari, 2002; Westermann, 2008; Klecker and Westermann, 2021).

**FIGURE 3:**
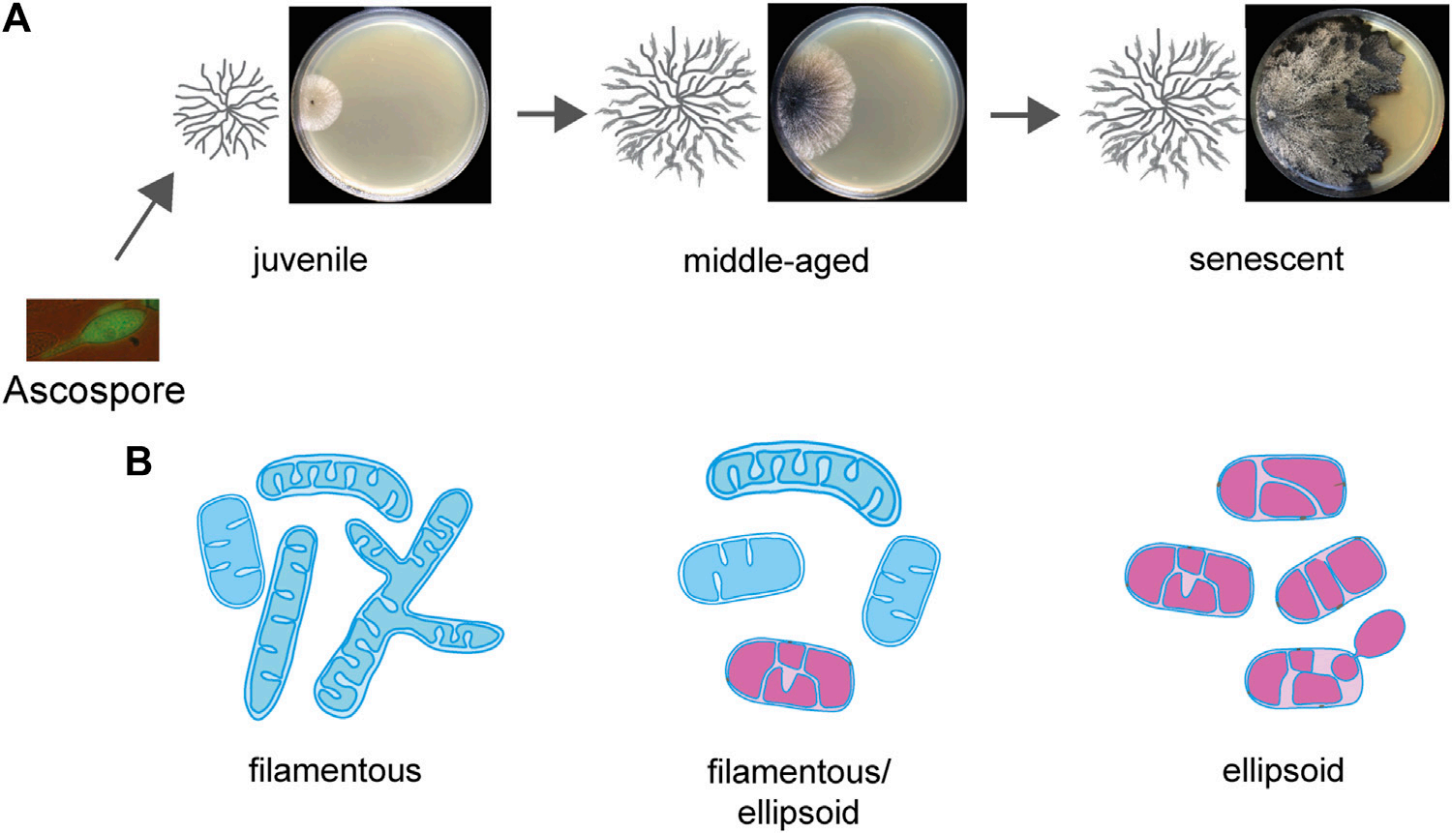
Age-related alterations of mitochondrial morphology during aging of *Podospora anserina*. **(A)** Morphology of an aging *Podospora anserina* culture grown on a solid medium. **(B)** Age-related changes in mitochondria. In juvenile cultures, the mitochondrial morphotype is filamentous, containing tubular cristae. In senescent mitochondria, the morphotype is predominantly ellipsoid and contains a vesicular IMM system. Fully functional mitochondria are indicated in blue, and functionally impaired mitochondria are indicated in magenta.

In *P. anserina*, it was found that the mitochondrial morphology changes during aging (Figure 3) from filamentous to ellipsoid. The latter morphotype accumulates in old age as a result of increased fission resulting from an increased expression of the *PaDnm1* gene, which encodes the mitochondrial dynamin-like protein PaDNM1, a homolog of human DRP1. The deletion of *PaDnm1* revealed that the process of fission is strongly delayed and the mitochondria remain filamentous most of the time during the lifespan of the mutant. Only in senescent cultures, a PaDNM1-independent fragmentation occurs. As indicated by no differences in the growth rate and fertility, the fitness of the mutant is not affected, demonstrating that it is the healthy period of the lifetime, the healthspan, which is extended from 22 days of the wild type to 244 days in the mutant (Scheckhuber et al., 2007). This healthspan extension occurs along with a decrease in cellular ROS generation and a delayed release of hydrogen peroxide from the mycelium to the growth medium. Moreover, the *PaDnm1*-deleted mutant was found to be more resistant to the induction of apoptosis by the apoptosis elicitor etoposide. The observed effects on mitochondrial morphology and lifespan in *P. anserina* were also observed in a *Dnm1*-deleted mutant of *S. cerevisiae* (Scheckhuber et al., 2007). The impact of the mitochondrial dynamics on the aging of *P. anserina* was further supported by a subsequent study in which the abundance of other fusion and fission proteins was increased (Scheckhuber et al., 2008). Complementary to the lifespan-extending effect of the *PaDnm1*-deleted strain, the overexpression of this gene had a lifespan-shortening effect. Moreover, while the overexpression of another fission gene, *PaFis1*, did not show any effect on the lifespan, the double-mutant overexpressing *PaDnm1* and *PaFis*1 led to a reduction of the lifespan beyond that of the *PaDnm1* overexpressor. However, in the overexpression study, the overexpression of another potential fission gene, *PaMdv1*, and the fusion gene *PaFzo1* did not show any effect on the lifespan although the mitochondrial morphology was changed.

## 5 Membrane trafficking and autophagy

**FIGURE 4:**
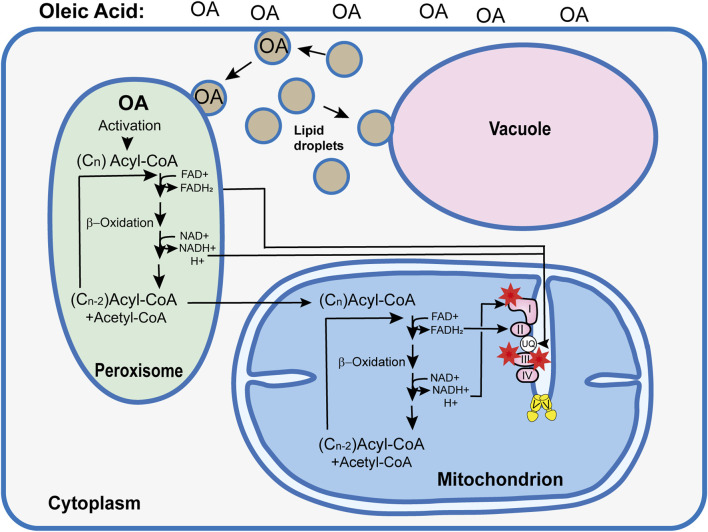
Simplified model explaining the consequences of an oleic acid (OA) diet on the *Podospora anserina* wild type. Use of oleic acid (C_18_H_34_O_2_) as the sole carbon source requires the metabolization of this long-chain fatty acid in peroxisome via ß-oxidation. In subsequent reaction cycles, this fatty acid is broken down to acetyl-Co and acyl-CoA. During this process, FADH_2_ and NADH+ H^+^ are formed, which, most likely via shuttle systems (not shown) (Wanders et al., 2015), are transported into the mitochondrial RC, where they contribute to ATP and superoxide (red asterisks) generation. Short acyl-CoA molecules enter the mitochondria, where they are further metabolized. In contrast to the NADH imported into the mitochondria that enters the RC at the ubiquinol (UQ) pool, NADH generated by mitochondrial ß-oxidation transfers electrons to mitochondrial complex I, increasing the risk of superoxide production. In *Podospora anserina*, an OA diet induces the formation of lipid droplets. These organelles transport OA to the peroxisome. In addition, they are active in maintaining membrane homeostasis via the transport of phospholipids to the vacuole membrane. This function became apparent from the analysis of the *PaAtg1*-deleted mutant, which was affected in membrane trafficking and in vacuolar function and autophagy (Henkel et al., 2020; Schürmanns et al., 2022).

Another important role of biomembranes with a strong impact on aging is related to autophagy, the controlled degradation of surplus or damaged cellular components by the lysosome or the vacuole. In *P. anserina*, it is the vacuole, as a cellular compartment enclosed by a single phospholipid bilayer, that performs this function. After induction, cellular components designated for degradation are either directly taken up via invaginations of the vacuolar membrane (microautophagy) or by the fusion of cargo-containing vesicles, termed autophagosomes, with the vacuolar membrane (macroautophagy). In *P. anserina*, autophagy is a “longevity assurance mechanism” because its inactivation via the deletion of essential genes controlling the pathway leads to a lifespan reduction (Knuppertz et al., 2014; Henkel et al., 2020). One such gene is *PaAtg24* coding for the sorting nexin PaATG24, a homolog of human SNX4. Sorting nexins are known to be involved in vesicle transport, membrane trafficking, and protein sorting. The ablation of PaATG24 impacts the morphology and size of the vacuoles, which are much smaller than those in the wild type. Moreover, non-selective (general autophagy) and selective autophagy of peroxisomes (pexophagy) and mitochondria (mitophagy) are also affected. General autophagy and pexophagy are almost completely blocked, and mitophagy is slightly decreased. During aging, a PaATG24-independent form of mitophagy is induced, which, however, appears not to be efficient enough to remove the dysfunctional mitochondria to prevent aging (Henkel et al., 2020). Subsequently, it was found that the ablation of the sorting nexin leads to mislocalization of PaSNC1, a “vesicle-associated receptor protein” (v-SNARE) that plays a key role in proper membrane–vesicle fusion (Ma et al., 2017). As a consequence, endosomes accumulate in the cytosol, membranes needed for the vacuole proliferation become limited, and the vacuole size and the efficiency of autophagy are strongly reduced. This scenario is supported by experiments in which *P. anserina* wild-type and mutant strains were grown on a medium in which the fermentable carbon source dextrin, which is metabolized by glycolysis and the Krebs cycle, was replaced by oleic acid. Metabolization of his carbon source requires peroxisomes (Figure 4). Most interestingly, an oleic acid diet led to a mean lifespan extension of the wild type from 24 to 30 days and from 19 to 37 days of the *PaAtg24*-deleted mutant (Schürmanns et al., 2022). This effect occurs along with the formation of lipid droplets, which are used for oleate uptake and its transport to peroxisomes for metabolization. Lipid droplets may also fuse with the vacuole, providing membranes for the proliferation of this organelle to the size of the wild-type vacuoles. As a consequence, efficient autophagy, as an important quality-control pathway, is restored in the mutant, contributing to the lifespan extension of the *PaAtg24*-deleted mutant, which is short-lived when grown on a standard growth medium with fermentable carbon sources.

## 6 Metabolic changes induced by oleic acid

The induction of lipid droplets by oleic acid occurs not only in the *PaAtg24*-deleted mutant but also in the wild type and is correlated with a lifespan extension (Schürmanns, 2022; Schürmanns et al., 2022). Apart from the effect of making biomembranes available for vacuole proliferation, an altered bioenergetic metabolism starting with ß-oxidation in peroxisomes is followed by the transfer of peroxisomal FADH_2_ and NADH, most likely via specific membrane-bound protein complexes (not shown), to mitochondria (Wanders et al., 2015) and feeding electrons into the RC (Figure 4). In contrast to NADH generated inside mitochondria, which transfers electrons to complex I of the RC, electron transfer of NADH from peroxisomes has to be imported in the IMS and, subsequently, using an alternative external NADH dehydrogenase to the ubiquinol pool in the IMM. In this way, complex I as a major superoxide generator (Barja, 2013) is bypassed (Figure 4). In the *PaAtg24*-deleted mutant, hydrogen peroxide that is formed by superoxide dismutase was shown to be lower than that generated by the standard respiratory chain of the wild type. However, after partial metabolization of oleic acid, acyl-CoA breakdown products with six or fewer C-carbon atoms are further metabolized by mitochondrial ß-oxidation. Electrons from the resulting redox equivalents can then enter the RC at complex I, and superoxide can be generated at this complex (Jezek et al., 2023). However, compared to the utilization of fermentable carbon sources, this type of metabolism is strongly reduced when the strains are grown on oleic acid as the sole carbon source. Under these conditions, hydrogen peroxide secretion as a measure of the cellular ROS load is strongly decreased (Schürmanns et al., 2022).

Overall, metabolic changes, as well as restoration of membrane trafficking and vacuole formation leading to a restoration of autophagy efficiency, contribute to the pronounced lifespan extension of the *PaAtg24*-deleted mutant, which, grown on a standard growth medium with fermentable carbon sources, is short-lived. Moreover, the observed lifespan of the wild type grown on oleic acid demonstrates a strong beneficial impact of this nutrient on aging.

## 7 Conclusion

In *P. anserina*, aging has a strong mitochondrial etiology. In old age, the efficient reorganization of the mtDNA biosynthesis of mitochondria is blocked and functional mitochondria cannot be sufficiently delivered to the growing parts of the vegetation body. This leads to growth stop and PCD. There are various pathways (e.g., ROS scavenging, mitochondrial dynamics, and degradation of damaged compounds) that can delay this process of degeneration, leading to longevity. Recent investigations emphasized the crucial relevance of biomembranes in such processes, which was already indicated in early work, demonstrating the important role of bioenergetic processes at the IMM. Specifically, the role of membrane trafficking, vacuoles, and autophagy underlined the strong impact of biomembranes. Furthermore, the impact of oleic acid on the longevity of *P. anserina* is striking because olive oil, which is rich in oleic acid, is a prominent component in Mediterranean diets, which is known to be beneficial for human health and successful aging (Trichopoulou and Dilis, 2007). At the mechanistic level, this positive effect results from reduced metabolic stress combined with effective cellular remodeling, which strongly depend on the biomembrane function and dynamics.
